# Knowledge and Attitudes of Undergraduate University Students in Oman With Regard to People Living With HIV

**DOI:** 10.7759/cureus.67006

**Published:** 2024-08-16

**Authors:** Ruba Al Bahri, Aaisha Al-Balushi, Abdullah Balkhair

**Affiliations:** 1 College of Medicine and Health Sciences, Sultan Qaboos University, Muscat, OMN; 2 Department of Medicine/Infectious Diseases Unit, Sultan Qaboos University Hospital, Muscat, OMN

**Keywords:** oman, university, students, stigma, attitude, knowledge, hiv

## Abstract

Background: There is scarce literature from the region pertinent to university students’ HIV-related knowledge, perception, attitudes, and behavior toward people living with HIV. Moreover, university students in Oman are remarkably uninformed about HIV, resulting in misconceptions and stigmatization among students.

Objective: This research aimed to examine HIV-related knowledge and attitudes of undergraduate medical and non-medical university students toward people living with HIV in Oman.

Methods:This was a qualitative cross-sectional study using convenience sampling to recruit participants from nine colleges at Sultan Qaboos University, Muscat, Oman. An online questionnaire composed of 17 Likert scale statements examining students’ knowledge and nine Likert scale statements exploring students’ attitudes was used. A knowledge score ≥ the mean was considered good knowledge, whereas a stigma score > the mean was regarded as stigmatization. A sample size of 376 students was computed using a Raosoft calculator (Raosoft, Inc., Seattle, Washington, United States) with a confidence level of 95% and a margin of error of 5%. Cronbach’s alpha for the 26-item questionnaire was α = 0.716. Responses were collected and analyzed using IBM SPSS Statistics for Windows, Version 26, (Released 2019; IBM Corp., Armonk, New York, United States). The questionnaire and the study protocol were approved by the institution’s medical research and ethics committee.

Results: A total of 678 undergraduate university students responded to the questionnaire including 450 (66.4%) and 228 (33.6%) female and male students, respectively. Medical students represented 20.8% of the responders. The mean knowledge score was 12.3 ± 1 signifying good knowledge in 72% of the students and the mean stigma score was 6.03 ± 3.51 indicating that 43.4% of the responders had a negative and stigmatizing attitude. Medical students had the highest mean knowledge score (14.2 ± 1.8) denoting good knowledge in 83.5% of the students. Additionally, medical students had the lowest mean stigma score (4.64 ± 3.32) implying that 29% of the surveyed medical students had negative attitudes toward people living with HIV. No significant association was found between students’ academic performance or students’ sex with mean knowledge scores. Contrarily, male sex was found to be significantly associated with lower mean stigma scores. No significant relationship between students’ knowledge scores and stigma scores was observed.

Conclusion: The findings of this study denote a substantial gap in HIV-related knowledge among university students, leading to undesirable attitudes toward people living with HIV. These findings call for an urgent need to escalate HIV awareness and educational programs tailored to university students in Oman.

## Introduction

HIV poses an enormous infectious threat to global health. According to the WHO, an estimated 39.9 million people were living with HIV at the end of 2023, of which 38.6 million were adults. Furthermore, an estimated 1.3 million people acquired HIV in 2023 and 630,000 people died from HIV-related causes globally in 2023 [1]. Nonetheless, the availability of antiretroviral therapy for treating and preventing HIV infection has shifted infection with HIV from a fatal illness into a controllable chronic disease [2].

According to the Joint United Nations Program on HIV/AIDS (UNAIDS) 2023 report, 2800 adults and children were estimated to be living with HIV in Oman in 2023, and the incidence of HIV was 0.04/1000 people (adults 15-49) [3]. It is interesting to note that only 85% of HIV-infected people in Oman know their HIV status according to the UNAIDS report [3]. In 2023, 221 new cases of HIV infections were recorded, of which 97% were attributed to sexual exposure [4]. This number of new cases is consistent with the rising trend of newly diagnosed HIV cases in Oman over the past five years, notably in the age group of 25-34 years, comprising 46% of the newly infected adults in 2021 [5]. The mounting trend of new HIV cases in individuals aged 25-34 years may possibly suggest changing behavior in this cohort coupled with a knowledge gap in HIV prevention strategies, among others.

A systematic review and meta-analysis of HIV knowledge and attitudes in the Arabian Peninsula reported an overall knowledge of 74.4% [6]. However, 52.8% of the surveyed individuals revealed negative attitudes toward people living with HIV [6]. Negative attitudes towards people living with HIV such as refusing casual contact with someone with HIV or socially isolating a member of a community because they have HIV are examples of stigmatizing attitudes, which can result in discriminatory behaviors with negative impacts on people living with HIV. A study by Al-Jabri et al. examined the knowledge, attitude, and behavior toward HIV testing and self-protection in a cohort of 1000 pregnant Omani women and found that a higher level of knowledge on HIV was significantly associated with a favorable behavior related to voluntary testing, disclosure, and seeking professional assistance in the event of a positive HIV test [7].

University students in the region are remarkably uninformed about HIV as shown by several studies [8-10]. A study that examined the attitudes of undergraduate university students in Oman toward people living with HIV showed an unacceptable level of misconception and stigmatization among the students. Just over 50% of the surveyed students reported in the affirmative to attend a class with a fellow student with HIV, and 65% of the students reported an unwillingness to care for a family member with HIV [8]. In a cross-sectional survey of more than 2000 students from four universities in the United Arab Emirates, exploring knowledge and attitudes toward HIV, the overall average knowledge score was 61%, while 85% of students expressed negative attitudes toward people living with HIV [9]. A similar study from Saudi Arabia examining medical students’ knowledge, attitudes, and beliefs about HIV reported mean HIV knowledge and attitude scores of 64.5% and 67.5%, respectively, with more than half exhibiting ignorance about some modes of transmission, and 81% of the students stated that they would not visit the homes of friends with HIV-infected members [10].

This study aimed to examine HIV-related knowledge and attitudes of undergraduate medical and non-medical university students toward people living with HIV in Oman.

## Materials and methods

Study objectives

This study had four objectives: 1) to examine HIV-related knowledge and attitudes of undergraduate university students, 2) to compare knowledge and stigma scores amongst students from nine colleges of the same university, 3) to examine the relationship between students’ academic performance and their knowledge and stigma scores, and 4) to explore the relationship between students’ HIV-related knowledge and stigma scores.

Study design

This is a qualitative cross-sectional study to assess the knowledge and attitude of medical and non-medical university undergraduate students towards HIV. The study utilized a non-probability convenience sample to recruit participants from nine colleges at Sultan Qaboos University, Muscat, Oman, from September 1 to November 30, 2023.

Sample size

The minimum sample size was computed using a Raosoft calculator (Raosoft, Inc., Seattle, Washington, United States) with a confidence level of 95% and a margin of error of 5%. Based on the number of undergraduate students at Sultan Qaboos University in 2022 (16,931 students), a minimum sample size of 376 responders from both sexes and from all nine colleges was assumed. All eligible responders between September 1 and November 30, 2023, were included in the analysis.

Inclusion and exclusion criteria

Undergraduate students of Sultan Qaboos University from all nine colleges and of any academic year were invited to participate in the study. Postgraduate students and university faculty and staff were excluded from the survey.

The questionnaire

The questionnaire was adapted from previous similar relevant studies [8,11,12] with some modifications made by local experts in HIV and public health (including the corresponding author) to suit local culture. Internal validation of the questionnaire (Cronbach’s alpha of 0.716) was done by an expert in biostatistics (see Acknowledgments).

The questionnaire consisted of a total of 26 statements using a three-point Likert scale and was composed of three parts: the first section was on demographic characteristics including age, sex, college, academic program, academic year, and student’s grade point average (GPA); the second section consisted of 17 statements on knowledge including five on general knowledge, eight on knowledge on transmission, and four on knowledge on prevention; and the third section was on attitudes toward people living with HIV, and was composed of nine statements.

Validation of the questionnaire

Since the used questionnaire was not previously validated, a pilot study to validate the questionnaire targeting 25% (92 undergraduate university students) of the actual sample size was conducted. However, 139 students representing all nine colleges responded to the draft questionnaire. The piloted questionnaire consisted of 27 statements and the value for Cronbach’s alpha for the piloted questionnaire was α = 0.690. After the removal of one statement from the section on attitudes toward people living with HIV, the value for Cronbach’s alpha for the 26-item questionnaire was α = 0.716.

Consent

Participants were presented with a detailed information sheet (see Appendices). This information sheet includes a statement where participants can choose to opt out. Hence, implied informed consent was used where participants were informed that their consent was implied by submitting the completed questionnaire.

Data collection and scoring of responses

The questionnaire was structured using online Google Forms (Google LLC, Mountain View, California, United States) in Arabic and English languages using a dropdown question type. An invitation to participate was sent via a secured email link to undergraduate students from all nine colleges at Sultan Qaboos University. The questionnaire was sent to all eligible university students (target population=16,931 undergraduate students at the same university).

Responses were collected using Google Forms and were then imported to IBM SPSS Statistics for Windows, Version 26, (Released 2019; IBM Corp., Armonk, New York, United States). Responses to any of the 17 statements examining students’ knowledge were either “Yes,” “No,” or “I don’t know,” whereas responses to the nine statements exploring students’ attitudes toward people living with HIV used a multiple-choice grid with the options being any of the following: “agree,” “neutral,” or “disagree.” A correctly answered statement in the knowledge section of the questionnaire was credited one point, whereas an incorrect response or “I don’t know” response scored zero, making a total maximum knowledge score of 17 points (the higher the score, the higher the knowledge). Furthermore, a correctly answered statement in the attitude section of the questionnaire was given zero marks, an incorrectly answered statement was allocated two marks, and a neutral response was allotted one mark. The sum of these scores was referred to as the stigma score and could range from 0 to 18. The higher the score was, the higher the level of the stigma. A knowledge score ≥ the mean was considered good knowledge, while a score < the mean was considered insufficient knowledge. Similarly, a stigma score > the mean was regarded as stigmatization toward people living with HIV.

Statistical analysis

Data were analyzed using SPSS software version 26. Quantitative variables were expressed as mean ± standard deviation or median and were compared using a two-sample t-test or Mann-Whitney test, depending on whether they were normally distributed or not. Qualitative variables were expressed as percentages. Chi-square was used to examine the relationship between categorical variables and analysis of variance (ANOVA) or Kruskal-Walli’s test was used to compare means between groups. Pearson's correlation coefficient was used to examine the association between HIV knowledge scores and HIV stigma scores. The significance level for statistical testing was defined as two-tailed p < 0.05.

Ethical approval

The questionnaire and the study protocol were approved by the Medical Research and Ethics Committee of the College of Medicine and Health Sciences, Sultan Qaboos University, Oman (approval no.: MREC#3093). A participant information sheet in Arabic and in English was sent to students and informed consent was obtained. All data was anonymized.

## Results

**Figure 1:**
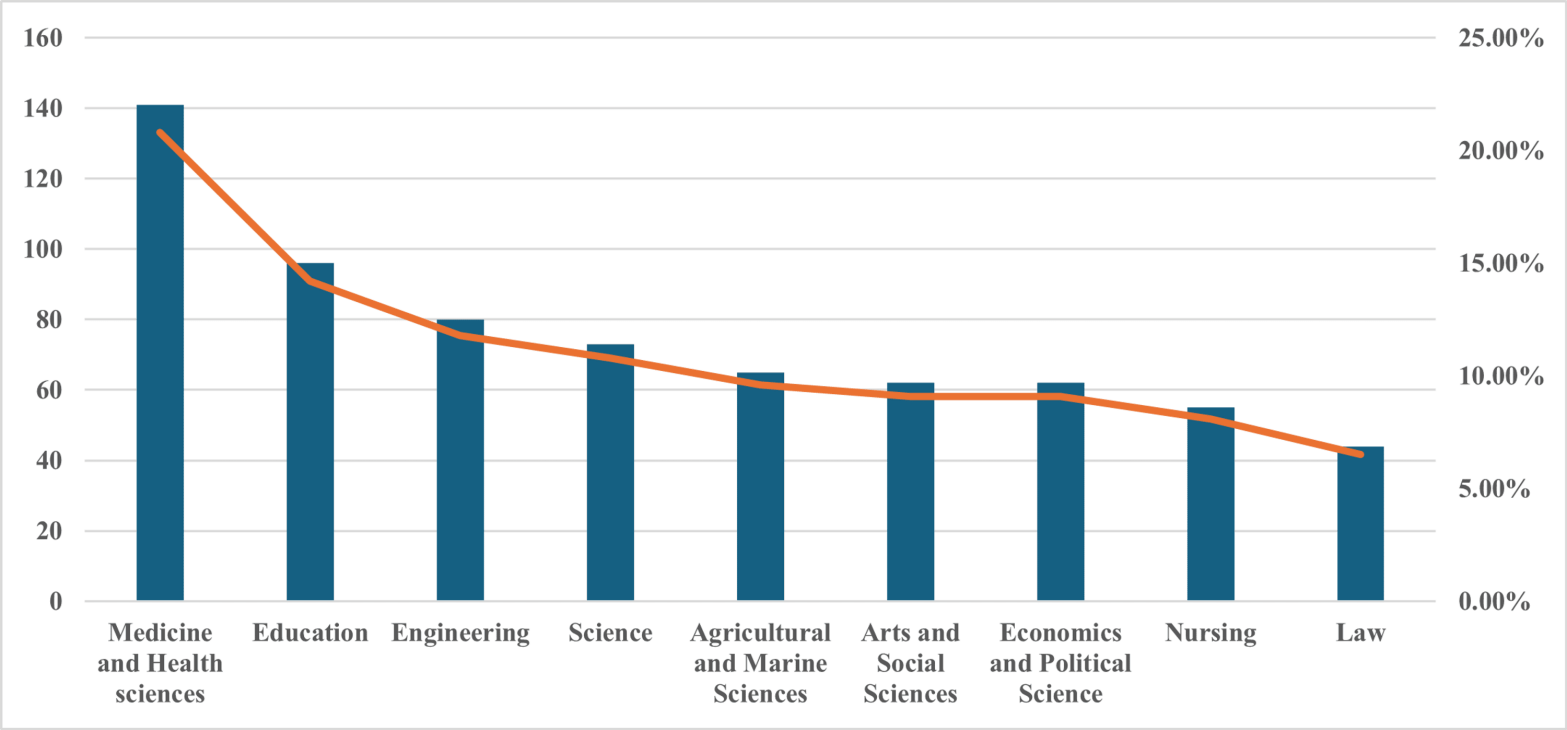
Study participants stratified by college

A total of 678 undergraduate university students (4%) responded to the questionnaire including 450 (66.4%) and 228 (33.6%) female and male students, respectively. A total of 340 (50.1%) of the participants had a GPA < 3.0 while 244 (35.9%) had a GPA ≥ 3.0. Data on GPA were missing for 94 students (13.9%) as they were first-year students (Table 1). Medical students (n = 141) represented 20.8% of the responders, while nursing students (n = 55) constituted 8.1% of those surveyed. Students from the remaining seven colleges (n = 482) comprised 71.1% of the responders (Figure 1).

**Table 1:** Baseline characteristics of the surveyed students. GPA: grade point average

	Description	Number	%
Sex	Male	228	33.6%
	Female	450	66.4%
GPA	GPA<3.0	340	50.1%
	GPA≥3.0	244	35.9%
	GPA not reported	94	13.7%
College (academic enrollment)	Medicine	141	20.8%
	Nursing	55	8.1%
	Other colleges	482	71.1%

Responses to statements on general knowledge (statements one to five)

A total of 93.4% of the surveyed students believed that HIV was primarily a sexually transmitted infection, while only 65% of the students knew that people living with HIV could be asymptomatic, and 54.3% believed that HIV could be cured. A total of 61.2% of the participants knew that treatment for HIV is currently life-long. Regarding the fifth statement on general knowledge, “HIV is rare in Oman and only a few cases have been reported,” 56.2% of the surveyed students believed so (Table 2).

**Table 2:** Responses to the knowledge section of the questionnaire

Questionnaire knowledge statement	% of the students with a “YES” response to the statement
General knowledge (statements 1-5)	
1. HIV is primarily sexually transmitted	93.4%
2. People living with HIV might not have symptoms of the disease	65.0%
3. HIV can be completely cured	54.3%
4. People living with HIV require life-long treatment	61.2%
5. HIV is rare in Oman only a few cases have been reported	56.2%
Transmission (statements 6-13)	
6. HIV can be spread through coughing and sneezing	25.8%
7. HIV can be transmitted from mother to her baby	80.1%
8. HIV can be spread through a handshake with an infected person	11.8%
9. HIV can be spread through sharing needles	89.7%
10. HIV can be spread through sharing food with an infected person	73.2%
11. HIV can be transmitted through blood transfusion	93.7%
12. HIV can be transmitted through an open wound	55.9%
13. HIV can be transmitted through sexual intercourse	95.6%
Prevention (statements 14-17)	
14. Infection with HIV can be prevented by avoiding mosquito bite	43.8%
15. Infection with HIV can be prevented by not sharing needles	88.6%
16. Condoms offer complete protection against HIV	45.1%
17. Infection with HIV can be prevented by behaving in accordance with our religious morals	92.5%

Responses to statements on transmission (statements six to 13)

A total of 25.8% of the students believed that HIV could be spread through coughing and sneezing, 11.8% thought that HIV could be spread through handshakes with an infected person, and 73.2% contemplated that HIV could be transmitted through sharing food with an infected person. A total of 95.6% of students agreed that HIV was primarily transmitted through sexual intercourse, whereas 89.7% believed that HIV could be spread through sharing needles. A total of 93.7% thought that HIV could be transmitted through blood transfusion, 80.1% considered that HIV could be transmitted from a mother to her baby, and 55.9% thought that HIV could be transmitted through direct contact with an open wound (Table 2).

Responses to statements on prevention (statements 14-17)

A total of 43.8% of the surveyed students believe that infection with HIV could be prevented by avoiding mosquito bites, and 45.1% thought that condoms offered complete protection against HIV. A total of 88.6% of participants believed that infection with HIV could be prevented by not sharing needles (Table 2).

The mean knowledge score for the surveyed 678 students was 12.3 ± 1 on a scale of 17, equivalent to an average knowledge of 72% (66.5%-78.2%). Medical students and nursing students had mean knowledge scores of 14.2 ± 1.8 and 13.8 ± 2.0, respectively. The mean knowledge score for students from the remaining seven colleges was 11.8 ± 2.8. Students from the College of Law had the highest knowledge score among the non-health sciences colleges with a knowledge score of 12.3, whereas participants from the College of Arts and Social Sciences attained the lowest score of 11.4. The difference in knowledge score and student college enrollment was statistically significant (p < 0.0001). The mean knowledge scores for students from all nine colleges are depicted in (Table 3).

**Table 3:** Mean knowledge score for participating students stratified by college

College	Mean knowledge score
Medicine and Health sciences	14.2
Nursing	13.8
Law	12.3
Engineering	12.2
Science	12.2
Education	11.7
Agricultural and Marine Sciences	11.5
Economics and Political Science	11.5
Arts and Social Sciences	11.4

Mean knowledge scores for male and female students were 12.6 ± 2.8 and 12.4 ± 3.0, respectively (p = 0.453). Students with a GPA ≥ 3.0 had an average knowledge score of 11.9 ± 2.8, and students with a GPA < 3.0 attained a mean knowledge score of 11.4 ± 3.0 (p = 0.874).

Responses to statements on attitudes toward people living with HIV (statements 18-26)

A total of 63% of the surveyed students believed that students living with HIV should not mix with other students while 32% of the students thought that if a family member contracted HIV, he/she should move out of the house. A total of 51% of the participants said that they should not play sports with fellow students living with HIV, and 90% of the surveyed students believed that people living with HIV should not get married (Table 4). A total of 46% of responders would not mind having a student with HIV in their classroom, 50% thought it was OK to share meals with people living with HIV, and 58% were willing to do volunteer work for people living with HIV. A total of 85% of the students confirmed that they would get tested for HIV upon suspicion. A total of 91% of the students answered yes to the statement “HIV prevention awareness campaigns targeting university students are needed” (Table 4).

**Table 4:** Responses to the attitudes section of the questionnaire.

Questionnaire statements (18-26) on attitudes toward people living with HIV	% of the students with a “correct” response to the statements
18. Students living with HIV should not mix with other students	37%
19. I would not mind having a student with HIV in my classroom	46%
20. I am willing to do volunteer work for people living with HIV	58%
21. If a family member contracts HIV, he/she should move out of the house	68%
22. I shall not play sports with people living with HIV	49%
23. People living with HIV should not get married	10%
24. It is OK to share meals with people living with HIV	50%
25. I would get tested if I thought I might have HIV	85%
26. HIV prevention awareness campaigns targeting university students are needed	91%

The mean stigma score for the surveyed 678 students was 6.03 ± 3.51. Medical students and nursing students had mean stigma scores of 4.64 ±3.32 and 6.16 ± 3.43, respectively. The mean stigma score for students from the remaining seven colleges was 6.42 ± 3.48. Students from the College of Engineering had the lowest stigma score after the medical students with a stigma score of 5.80; however, participants from the College of Education attained the highest stigma score of 7.11. The difference in stigma score and student college enrollment was statistically significant (p < 0.05). The mean stigma scores for students from all nine colleges are depicted in Table 5.

**Table 5:** Mean stigma score for participating students stratified by college

College	Mean stigma score
Medicine and Health sciences	4.64
Engineering	5.80
Science	6.07
Economics and Political Science	6.15
Nursing	6.16
Agricultural and Marine Sciences	6.46
Arts and Social Sciences	6.58
Law	6.68
Education	7.11

Mean stigma scores for male and female students were 5.63 ± 1.13 and 6.23 ± 0.81, respectively (p < 0.05) with a median score of six and a range of 0-16 for female students and a median score of 5 and a range of 0-14 for male students. A total of 205 female students (45.6%) had a stigma score > 6, while only 89 male students (39.1%) had a stigma score > 6. Students with a GPA ≥ 3.0 had an average stigma score of 5.89 ± 1.08, and students with a GPA < 3.0 attained a mean stigma score of 5.96 ± 0.92. No significant relationship between students’ knowledge and stigma scores was observed (Pearson's correlation coefficient (r) = 0.03226, r² = 0.001041, p = 0.402).

## Discussion

A recent bibliometric analysis of emerging trends in research on HIV among students found that the main target population of studies on HIV among students were medical and non-medical university students and showed that most cited articles on HIV among students were predominantly related to HIV knowledge, attitudes, and behaviors [13]. Notwithstanding this wealth of knowledge, there is a paucity of quality scientific literature from the region with regard to university students’ knowledge, perception, attitudes, and behavior toward people living with HIV, including their fellow students. Thus, this research endeavored to explore university students’ knowledge and their perceived attitude toward people living with HIV in Oman.

The present study identified a moderate level of knowledge among the surveyed university students with a mean knowledge score of 12.3 ± 1 on a scale of 17, signifying good knowledge in 72% of the students (range: 66.5%-78.2%). This level of knowledge, albeit inadequate in our assessment, is comparable with the finding of a systematic review from the region on HIV knowledge and attitudes with a reported overall knowledge of 74.4% [6]. It is important to highlight that reported HIV-related knowledge levels among university students vary widely and can be as low as 30% [14] and as high as > 80% [15,16] depending on the methodology used, characteristics of surveyed students, perceived knowledge adequacy, and locality, among other factors.

As shown in this study, surveyed students demonstrated severely limited HIV-related knowledge in four items related to transmission and prevention of HIV. This knowledge gap was evidenced by 25.8% of the students erroneously believing that HIV could be spread through coughing and sneezing, 11.8% thinking that HIV could be spread through a handshake with an infected person, 73.2% contemplating that HIV could be transmitted through sharing food with an infected person, and 45.1% thinking that condoms offered complete protection against HIV. An additional point of concern is that ~50% of the students mistakenly perceived that HIV was rare in Oman and only a few cases had been reported. These findings denote a substantial gap in HIV-related knowledge among the surveyed students and call for an urgent need to escalate HIV awareness and address this consequential inadequacy in knowledge.

Albeit predictable, medical and nursing students had the highest knowledge scores amongst students from all nine colleges with average knowledge levels of 83.5% and 81.2%, respectively. This knowledge contrast between health sciences students and other students is possibly due to academia rather than outreach awareness campaigns and public health initiatives. A study assessing HIV knowledge and practices among 3,727 undergraduate students including 2,539 medical students in 10 African countries found that medical students had significantly higher HIV-related knowledge than non-medical students at 48% [17]; albeit, this is remarkably lower than the performance of medical students in the present study. A study from Saudi Arabia including 253 medical students and 349 non-medical students showed that medical students had the highest level of HIV-related knowledge [11]. Furthermore, two studies exclusively comprised of medical students from Jordan and Saudi Arabia reported an average knowledge of 71.15% and 64.5%, respectively [12, 10]. We believe that this satisfactory performance of the surveyed medical students in the current study reflects the comprehensiveness of HIV-related education in the current medical curriculum.

It is intriguing to note that the present study showed that neither student academic performance as measured by GPA nor students’ sex had any significant impact on knowledge scores. This finding is consistent with the findings from a study from India which showed no association between students' gender and knowledge [15]. It nevertheless contrasts with the finding from a large Chinese study that showed a significant correlation between levels of students’ HIV-related knowledge and their gender and academic grade [16].

Likewise, this study shows that the attitudes of a substantial number of university students toward people living with HIV were negative, reflecting an elevated level of stigma. The CDC defines HIV stigma as negative attitudes and beliefs about people with HIV [18]. The present study found a troubling level of stigma amongst university students, evidenced by an average stigma score of ~ 6 points on a scale of 18, corresponding to a perceived negative attitude toward people living with HIV in 43.4% of the responders. This adversely perceived attitude was particularly pronounced in response to the following statements in the questionnaire: “People living with HIV should not get married,” “Students living with HIV should not mix with other students,” and “I would mind having a student with HIV in my classroom,” where 90%, 63%, and 54%, respectively, incorrectly agreed with the statements. Sadly, the overwhelmingly erroneous responses to these three statements present discriminatory behaviors and incorrectly endorse the social isolation of people, including fellow university students, living with HIV.

With the exception of a single study from Jordan that was exclusively conducted among medical students [12], four other studies amongst heterogeneous groups of university students from the Arabian peninsula, Iran, and India demonstrated higher rates (range: 46.38%-85%) of negative attitudes toward people living with HIV [6,9,14,15]. A study by James et al. examined the relationship between HIV testing history and stigma in university students and demonstrated that stigma was one of the strongest barriers to HIV testing and treatment [19]. Unfortunately, 15% of the students in the current study would not opt for testing even if they thought they might have HIV. We suspect that this negative attitude indicates the level of perceived stigma by the surveyed university students toward HIV. This level of unwillingness for testing amongst university students was documented in a similar survey involving 200 students from the same university two decades ago [8].

Although medical students outperformed students from other colleges in terms of attaining the lowest level of stigmatization, 29% of medical students demonstrated a negative attitude toward people living with HIV. This finding is both unexpected, largely concerning, and perhaps reflective of the shortfall of the medical curriculum among other reasons. This level of stigmatization among medical students toward people living with HIV was demonstrated by several studies including a study among 1,361 medical students in Jordan, a study of 204 medical students in Saudi Arabia, and a study among 171 medical students in Bosnia and Herzegovina with 33%, 32.5%, and 26.4% of participants, respectively, found to have negative attitudes toward people living with HIV [12,10,20]. It is interesting to note that, unlike their performance in knowledge, nursing students had a below-average stigma score and ranked fifth among the nine colleges. Since medical and nursing students are the future healthcare providers, their negative attitudes toward people living with HIV have foremost consequences; hence, interventions to reduce stigmatizing attitudes toward people living with HIV must be incorporated into medical and nursing training curricula [21].

Although students’ academic performance as reflected by GPA did not impact students’ attitudes, male students tended to have favorable attitudes toward people living with HIV as reflected by statistically significant lower average stigma score (5.63 vs. 6.23) when compared to female students. To the best of our knowledge, the association between students’ male sex and favorable attitudes toward people living with HIV was not observed in other studies. Contrarily, a study by Badahdah et al. found that female students held more positive attitudes toward people with HIV compared to male students [22], whereas a study by Khargekar et al. found no association between students' gender and attitude toward people living with HIV [15]. Although the present study did not show a relationship between students’ HIV-related knowledge and stigma scores, other studies have demonstrated conflicting results with a positive association in one study [16] but a negative correlation in another [11].

The present study has several limitations, in addition to those inherent in convenience sampling. First, it is a single-center qualitative research conducted in one university in one locality; hence, the findings may not be generalizable to students from other colleges and universities. Second, the study relied on self-reported data using a self-administered questionnaire; hence, responses may be imprecise. Third, is the use of a non-standardized questionnaire with the drawback of inadequate validation. Despite these limitations, this study had a clearly defined study population, had a large sample size with extensive representation from all colleges including medical and nursing students, and used a newly developed, locally adapted, and piloted online tool. We believe that the findings of this study will add to the scarce scientific literature on university students’ knowledge and attitudes toward HIV.

## Conclusions

The findings of this study denote a substantial gap in HIV-related knowledge among university students, leading to undesirable attitudes toward people living with HIV. These findings call for an urgent need to escalate HIV-targeted awareness programs, develop educational forums tailored for university students, and redesign the current medical and nursing curricula with the aim of improving students’ attitudes and reducing stigmatizing behaviors toward people living with HIV.

## Appendices

Participant Information Sheet

Study Title: Knowledge, Attitude, and Behavior of Medical and Non-Medical University Students Toward HIV

-----------------------------------------------------------------------------------------------------------------

You are invited to participate in a student-led research questionnaire examining the knowledge, attitude, and behavior of undergraduate students at Sultan Qaboos University toward HIV and toward people living with HIV. *Before you decide whether you wish to participate in this study*, it is important for you to understand why the research is being done and what it will involve. Please take the time to read the following information.

1.          What is the purpose of this study?

The purpose is to explore knowledge, attitudes, and behavior among undergraduate students at Sultan Qaboos University toward HIV and toward people living with HIV.

2.          Why have I been invited to participate in this study?

If you are an undergraduate student from any of the nine colleges, we are interested to know what you know about the subject as your participation will enrich the research outcome.

3.          What if I don’t want to take part in this study?

Participation in this study is voluntary. It is completely up to you whether or not you participate.

4.          What does this study involve?

If you agree to participate in this study, you will be asked to answer a questionnaire in an online Google Form through a secure link.

5.          Are there risks to me in taking part in this survey?

We perceive NO risk.

6.          Will I benefit from the study?

This research shall provide valuable insights into the knowledge, attitudes, and behavior of university students related to HIV, which can inform the development of targeted interventions and educational programs for university students (including you).

7.          Will taking part in this study cost me anything?

We believe that your participation in this study will not cost you anything. On the contrary, your participation shall be beneficial to you.

8.          How will my confidentiality be protected?

All data collected from participants will be kept strictly confidential and anonymous. The survey responses will be stored on a secure server that only the research team can access. Individual responses will not be identifiable.

9.          What happens with the results?

We plan to publish the results. In any publication, information will be provided in such a way that you cannot be identified.

Thank you for taking the time to consider this study.

*If you wish to take part in it, please answer the online questionnaire by opening the below link*.
